# Crystal structure of poly[[μ-1,1′-(butane-1,4-di­yl)bis­(1*H*-benzimidazole)-κ^2^ *N* ^3^:*N* ^3′^]{μ-4,4′-[1,4-phenyl­enebis(­oxy)]di­ben­zo­ato-κ^4^ *O*,*O*′:*O*′′,*O*′′′}cobalt(II)]

**DOI:** 10.1107/S2056989015008294

**Published:** 2015-05-20

**Authors:** Chen Xie, ChangGe Zheng

**Affiliations:** a School of Chemical and Material Engineering, Jiangnan University, 1800 Lihu Road, Wuxi, Jiangsu Province 214122, People’s Republic of China

**Keywords:** crystal structure, metal–organic frameworks, bis-benzimidazole, di­carboxyl­ate

## Abstract

In the title compound, [Co(C_20_H_12_O_6_)(C_18_H_18_N_4_)]_*n*_, the Co^II^ atom, located on a twofold rotation axis, is hexa­coordinated to four O from two bis-bidentate 4,4′-[phenyl­enebis(­oxy)]dibenzoate (*L*) ligands and two N atoms from two 1,1′-(butane-1,4-di­yl)bis­(1*H*-benzimidazole) (bbbm) ligands, forming a distorted octahedral *cis*-N_2_O_4_ coordination environment. Polymeric zigzag chains along [102] are built up by the bridging *L* ligands. These chains are additionally connected by the bbbm ligands to produce a two-dimensional coordination polymer parallel too (010).

## Related literature   

As a result of their intriguing variety of architectures and topologies, metal–organic frameworks (MOFs) with transition metal Co have received extensive inter­est. Bis-benzimidazole ligands bearing with butyl spacers are a good choice for the assembly of versatile entangled structures, see: Liu *et al.* (2008 ▸). Complexes with di­carboxyl­ate ligands represent the most reliable and typical building blocks which can be jointly applied to synthesize a wide range of compounds with coord­ination networks, see: Du *et al.* (2013 ▸). For the potential properties of metal–organic complexes involving polycarboxyl­ate ligands or bis-benzimidazole, see: Li *et al.* (2011 ▸); Wang *et al.* (2004 ▸); Sun *et al.* (2009 ▸); Wang *et al.* (2005 ▸); Łyszczek & Mazur (2012 ▸); Meng *et al.* (2003 ▸).
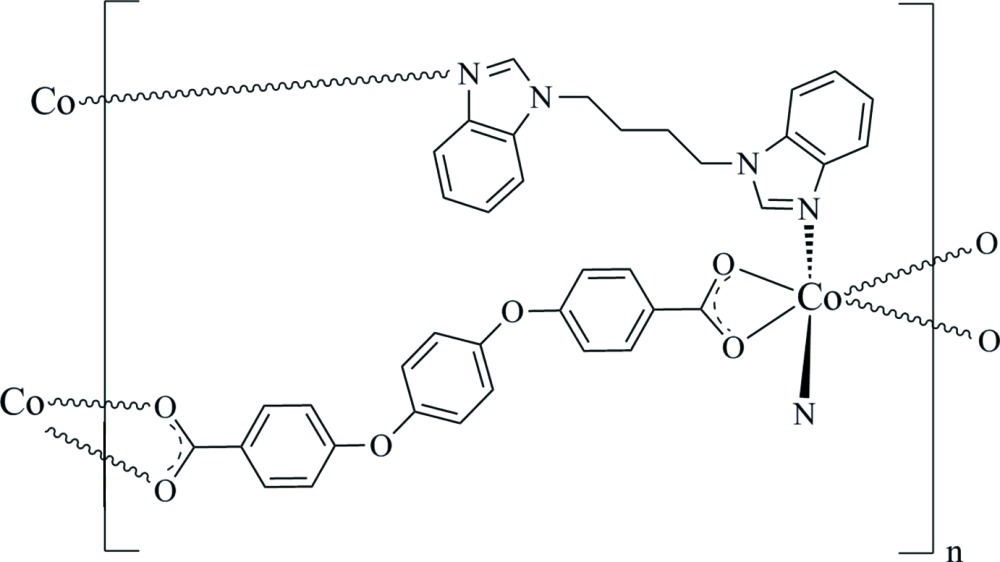


## Experimental   

### Crystal data  

[Co(C_20_H_12_O_6_)(C_18_H_18_N_4_)]
*M* *_r_* = 697.59
Monoclinic, 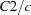

*a* = 16.961 (4) Å
*b* = 16.446 (3) Å
*c* = 12.987 (3) Å
β = 117.022 (3)°
*V* = 3227.1 (12) Å^3^
*Z* = 4
Mo *K*α radiation
μ = 0.59 mm^−1^
*T* = 296 K
0.27 × 0.24 × 0.19 mm

### Data collection  

Bruker APEXII CCD diffractometer
Absorption correction: multi-scan (*SADABS*; Bruker, 2007 ▸) *T*_min_ = 0.858, *T*_max_ = 0.897
7207 measured reflections
2836 independent reflections
2385 reflections with *I* > 2σ(*I*)
*R*_int_ = 0.052

### Refinement  

*R*[*F* ^2^ > 2σ(*F* ^2^)] = 0.049
*wR*(*F* ^2^) = 0.140
*S* = 1.02
2836 reflections
222 parameters
H-atom parameters constrained
Δρ_max_ = 0.61 e Å^−3^
Δρ_min_ = −0.65 e Å^−3^

### 

Data collection: *APEX2* (Bruker, 2007 ▸); cell refinement: *SAINT* (Bruker, 2007 ▸); data reduction: *SAINT*; program(s) used to solve structure: *SHELXS97* (Sheldrick, 2008 ▸); program(s) used to refine structure: *SHELXL97* (Sheldrick, 2008 ▸); molecular graphics: *SHELXTL* (Sheldrick, 2008 ▸); software used to prepare material for publication: *SHELXTL*.

## Supplementary Material

Crystal structure: contains datablock(s) I, New_Global_Publ_Block. DOI: 10.1107/S2056989015008294/im2463sup1.cif

Structure factors: contains datablock(s) I. DOI: 10.1107/S2056989015008294/im2463Isup2.hkl

**Fig. 1.**
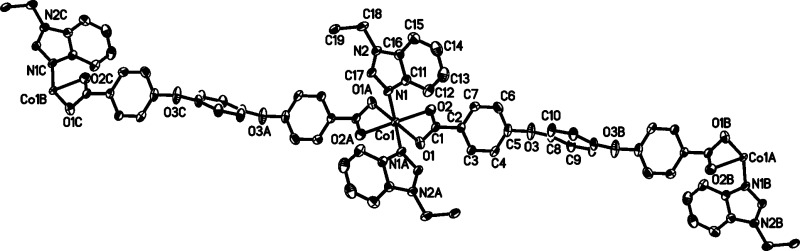
The molecular structure of [Co(C20H12O6)(C18H18N4)]n, with the non-H atom-numbering scheme and 30% probability displacement ellipsoids.

20 12 6 18 18 4 n . DOI: 10.1107/S2056989015008294/im2463fig1.tif
The mol­ecular structure of [Co(C_20_H_12_O_6_)(C_18_H_18_N_4_)]_*n*_, with the non-H atom-numbering scheme and 30% probability displacement ellipsoids.

**Fig. 2.**
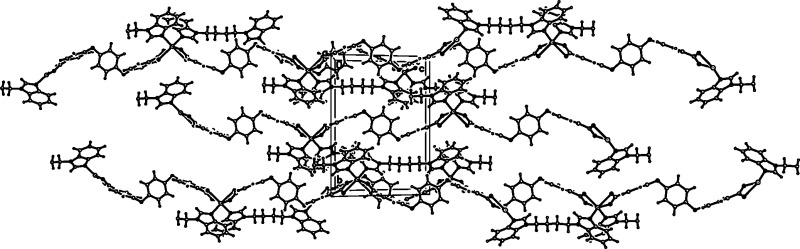
Three-dimensional network structure of [Co(C20H12O6)(C18H18N4)]n formed by C—H–O interaction.

20 12 6 18 18 4 n . DOI: 10.1107/S2056989015008294/im2463fig2.tif
Three-dimensional network structure of [Co(C_20_H_12_O_6_)(C_18_H_18_N_4_)]_*n*_ formed by C—H–O inter­action.

CCDC reference: 1045681

Additional supporting information:  crystallographic information; 3D view; checkCIF report

## supplementary crystallographic information

### S1. Comment

Because of the intriguing varieties of architectures and topologies, metal-organic frameworks (MOFs) with transition-metal Co have received extensive inter­ests. The bis-benzimidazole ligands bearing with butyl spacers are a good choice for the assembly of versatile entangled structures. (Ying-Ying Liu *et al.*, 2008) Complexes with the di­carboxyl­ate ligands represent the most reliable and typical building blocks which can be jointly applied to synthesize a wide range of desired coordination networks (Du *et al.*, 2013).

Single-crystal X-ray diffraction analyses reveal that Co(II) is six-coordinate. The asymmetric unit contains one Co(II) atom, a di­carboxyl­ate ligand and a bbbm ligand. Two carboxyl­ate groups adopt a chelating bidentate mode to connect one Co(II) atoms. The Co—O bond length is 2.3705 (24)Å (O1) and 2.0422 (21)Å (O2), the Co—N bond length is 2.0797 (26)Å.

### S2. Synthesis and crystallization

A mixture of 1,4-bis­(4-carboxyl­phen­oxy)­benzene (0.035 g, 0.1 mmol), 1,1'-(1,4-butyl) bis-benzimidazole (0.029 g, 0.1 mmol), Co(NO_3_)_2_ H_2_O (0.029 g, 0.1 mmol), and deionized water (9 mL) was stired for 10 min at ambient temperature. Then the mixture was sealed in a Teflon-lined stainless vessel(25 mL) and heated at 160 °C for 3 days. The vessel was cooled to 50 °C by 9 °C decrease per hour, then cooled to ambient temperature directly. Amaranth transparent block-like crystal were obtained by fitretion and washed with deionized water. Yield: 34.2 mg(49 %, based on Co) Elemental analysis (%) calcd. for CoC_38_H_30_N_4_O_6_: C 65.33, H 4.3, N 8.02. Found: C 65.38, H 4.39, N 8.11.

### S3. Refinement

The H atoms bonded to C atoms were introduced at calculated positions and refined using a riding model, with U_iso_(H) = 1.2U_eq_(C) and C–H distances of 0.93–0.97 Å.

### Crystal data

**Table:**

[Co(C_20_H_12_O_6_)(C_18_H_18_N_4_)]	*F*(000) = 1444
*M**_r_* = 697.59	*D*_x_ = 1.436 Mg m^−^^3^
Monoclinic, *C*2/*c*	Mo *K*α radiation, λ = 0.71073 Å
Hall symbol: -C 2yc	Cell parameters from 2720 reflections
*a* = 16.961 (4) Å	θ = 2.7–26.5°
*b* = 16.446 (3) Å	µ = 0.59 mm^−^^1^
*c* = 12.987 (3) Å	*T* = 296 K
β = 117.022 (3)°	Block, purple
*V* = 3227.1 (12) Å^3^	0.27 × 0.24 × 0.19 mm
*Z* = 4	

### Data collection

**Table:**

Bruker APEXII CCD diffractometer	2836 independent reflections
Radiation source: fine-focus sealed tube	2385 reflections with *I* > 2σ(*I*)
Graphite monochromator	*R*_int_ = 0.052
phi and ω scans	θ_max_ = 25.0°, θ_min_ = 1.8°
Absorption correction: multi-scan (*SADABS*; Bruker, 2007)	*h* = −15→20
*T*_min_ = 0.858, *T*_max_ = 0.897	*k* = −17→19
7207 measured reflections	*l* = −14→15

### Refinement

**Table:**

Refinement on *F*^2^	Primary atom site location: structure-invariant direct methods
Least-squares matrix: full	Secondary atom site location: difference Fourier map
*R*[*F*^2^ > 2σ(*F*^2^)] = 0.049	Hydrogen site location: inferred from neighbouring sites
*wR*(*F*^2^) = 0.140	H-atom parameters constrained
*S* = 1.02	*w* = 1/[σ^2^(*F*_o_^2^) + (0.0857*P*)^2^ + 2.0556*P*] where *P* = (*F*_o_^2^ + 2*F*_c_^2^)/3
2836 reflections	(Δ/σ)_max_ < 0.001
222 parameters	Δρ_max_ = 0.61 e Å^−^^3^
0 restraints	Δρ_min_ = −0.65 e Å^−^^3^

### Special details

**Table:**

Geometry. All e.s.d.'s (except the e.s.d. in the dihedral angle between two l.s. planes) are estimated using the full covariance matrix. The cell e.s.d.'s are taken into account individually in the estimation of e.s.d.'s in distances, angles and torsion angles; correlations between e.s.d.'s in cell parameters are only used when they are defined by crystal symmetry. An approximate (isotropic) treatment of cell e.s.d.'s is used for estimating e.s.d.'s involving l.s. planes.
Refinement. Refinement of *F*^2^ against ALL reflections. The weighted *R*-factor *wR* and goodness of fit *S* are based on *F*^2^, conventional *R*-factors *R* are based on *F*, with *F* set to zero for negative *F*^2^. The threshold expression of *F*^2^ > σ(*F*^2^) is used only for calculating *R*-factors(gt) *etc*. and is not relevant to the choice of reflections for refinement. *R*-factors based on *F*^2^ are statistically about twice as large as those based on *F*, and *R*- factors based on ALL data will be even larger.

### Fractional atomic coordinates and isotropic or equivalent isotropic displacement parameters (Å^2^)

**Table:**

	*x*	*y*	*z*	*U*_iso_*/*U*_eq_	
Co1	0.5000	0.10089 (3)	0.7500	0.0377 (2)	
N1	0.46215 (15)	0.17924 (14)	0.84527 (17)	0.0426 (5)	
O2	0.37200 (15)	0.07207 (16)	0.63915 (18)	0.0635 (6)	
O1	0.46556 (13)	0.00024 (14)	0.60520 (19)	0.0591 (6)	
N2	0.42235 (15)	0.21416 (15)	0.98059 (18)	0.0437 (5)	
C16	0.36476 (17)	0.25422 (16)	0.8810 (2)	0.0424 (6)	
C1	0.38817 (18)	0.02076 (17)	0.5798 (2)	0.0432 (6)	
C2	0.31148 (17)	−0.01457 (15)	0.4757 (2)	0.0378 (6)	
C3	0.32303 (18)	−0.03657 (17)	0.3804 (2)	0.0422 (6)	
H3	0.3786	−0.0317	0.3830	0.051*	
C17	0.47733 (18)	0.17001 (18)	0.9539 (2)	0.0438 (6)	
H17	0.5214	0.1365	1.0064	0.053*	
O3	0.10093 (17)	−0.10366 (14)	0.1800 (2)	0.0759 (8)	
C11	0.39104 (18)	0.23265 (16)	0.7975 (2)	0.0417 (6)	
C4	0.2531 (2)	−0.06546 (17)	0.2819 (2)	0.0477 (7)	
H4	0.2610	−0.0801	0.2181	0.057*	
C19	0.5047 (2)	0.2089 (2)	1.1943 (2)	0.0537 (7)	
H19A	0.5404	0.2550	1.1945	0.064*	
H19B	0.5351	0.1598	1.1908	0.064*	
C7	0.22884 (19)	−0.0219 (2)	0.4712 (2)	0.0512 (7)	
H7	0.2205	−0.0064	0.5344	0.061*	
C13	0.2759 (2)	0.3149 (2)	0.6626 (3)	0.0696 (10)	
H13	0.2448	0.3361	0.5885	0.084*	
C8	0.05289 (18)	−0.04860 (18)	0.0917 (2)	0.0508 (7)	
C10	0.0458 (2)	0.0333 (2)	0.1086 (2)	0.0576 (8)	
H10	0.0771	0.0557	0.1819	0.069*	
C5	0.1718 (2)	−0.07240 (18)	0.2787 (2)	0.0510 (7)	
C12	0.3458 (2)	0.26448 (19)	0.6859 (3)	0.0572 (8)	
H12	0.3628	0.2517	0.6291	0.069*	
C18	0.4176 (2)	0.2131 (2)	1.0912 (2)	0.0584 (8)	
H18A	0.3825	0.1667	1.0916	0.070*	
H18B	0.3873	0.2618	1.0961	0.070*	
C15	0.2935 (2)	0.30607 (19)	0.8577 (3)	0.0579 (8)	
H15	0.2766	0.3200	0.9142	0.069*	
C9	0.0075 (2)	−0.08197 (19)	−0.0168 (3)	0.0540 (8)	
H9	0.0127	−0.1371	−0.0282	0.065*	
C14	0.2497 (2)	0.3356 (2)	0.7465 (3)	0.0712 (10)	
H14	0.2015	0.3700	0.7270	0.085*	
C6	0.15846 (19)	−0.0521 (2)	0.3733 (3)	0.0591 (8)	
H6	0.1032	−0.0586	0.3708	0.071*	

### Atomic displacement parameters (Å^2^)

**Table:**

	*U*^11^	*U*^22^	*U*^33^	*U*^12^	*U*^13^	*U*^23^
Co1	0.0393 (3)	0.0508 (4)	0.0221 (3)	0.000	0.0133 (2)	0.000
N1	0.0524 (13)	0.0490 (13)	0.0281 (11)	0.0031 (10)	0.0198 (10)	−0.0016 (9)
O2	0.0606 (13)	0.0822 (16)	0.0457 (12)	−0.0111 (12)	0.0223 (10)	−0.0246 (11)
O1	0.0415 (11)	0.0632 (14)	0.0567 (13)	−0.0011 (10)	0.0083 (10)	−0.0031 (10)
N2	0.0495 (12)	0.0512 (14)	0.0342 (11)	−0.0018 (10)	0.0224 (10)	−0.0083 (10)
C16	0.0454 (14)	0.0390 (15)	0.0415 (14)	−0.0052 (12)	0.0185 (12)	−0.0062 (11)
C1	0.0465 (15)	0.0505 (16)	0.0281 (13)	0.0015 (12)	0.0130 (12)	0.0040 (11)
C2	0.0410 (13)	0.0369 (14)	0.0307 (13)	0.0026 (11)	0.0122 (11)	0.0039 (10)
C3	0.0439 (14)	0.0455 (15)	0.0342 (13)	0.0032 (12)	0.0152 (12)	0.0033 (11)
C17	0.0487 (15)	0.0527 (17)	0.0289 (13)	0.0023 (12)	0.0167 (11)	−0.0044 (11)
O3	0.0741 (16)	0.0512 (13)	0.0501 (13)	−0.0189 (11)	−0.0175 (12)	0.0059 (10)
C11	0.0491 (14)	0.0376 (14)	0.0340 (13)	−0.0043 (11)	0.0150 (12)	−0.0022 (10)
C4	0.0630 (18)	0.0448 (16)	0.0292 (13)	−0.0035 (14)	0.0155 (13)	0.0015 (11)
C19	0.0625 (18)	0.069 (2)	0.0373 (15)	−0.0063 (15)	0.0293 (14)	0.0003 (14)
C7	0.0501 (16)	0.065 (2)	0.0415 (15)	−0.0023 (14)	0.0236 (13)	−0.0036 (13)
C13	0.074 (2)	0.056 (2)	0.059 (2)	0.0119 (18)	0.0134 (18)	0.0121 (16)
C8	0.0381 (14)	0.0515 (18)	0.0407 (15)	−0.0122 (12)	−0.0016 (12)	0.0025 (12)
C10	0.0562 (17)	0.0547 (18)	0.0371 (15)	−0.0159 (15)	−0.0004 (13)	−0.0108 (13)
C5	0.0519 (16)	0.0420 (15)	0.0364 (15)	−0.0090 (13)	0.0001 (12)	0.0038 (12)
C12	0.0714 (19)	0.0513 (18)	0.0424 (16)	0.0000 (15)	0.0203 (15)	0.0041 (13)
C18	0.0693 (19)	0.076 (2)	0.0417 (16)	−0.0016 (17)	0.0352 (15)	−0.0120 (15)
C15	0.0546 (17)	0.0518 (18)	0.069 (2)	−0.0027 (14)	0.0294 (16)	−0.0109 (15)
C9	0.0506 (16)	0.0442 (16)	0.0478 (17)	−0.0096 (13)	0.0055 (14)	−0.0062 (13)
C14	0.0576 (19)	0.055 (2)	0.084 (3)	0.0104 (16)	0.0178 (19)	0.0052 (18)
C6	0.0381 (14)	0.068 (2)	0.064 (2)	−0.0069 (14)	0.0170 (14)	0.0044 (16)

### Geometric parameters (Å, º)

**Table:**

Co1—O2	2.042 (2)	C4—H4	0.9300
Co1—O2^i^	2.042 (2)	C19—C18	1.477 (4)
Co1—N1	2.080 (2)	C19—C19^ii^	1.526 (5)
Co1—N1^i^	2.080 (2)	C19—H19A	0.9700
Co1—O1	2.371 (2)	C19—H19B	0.9700
Co1—O1^i^	2.371 (2)	C7—C6	1.382 (4)
N1—C17	1.322 (3)	C7—H7	0.9300
N1—C11	1.390 (3)	C13—C12	1.363 (5)
O2—C1	1.255 (4)	C13—C14	1.392 (5)
O1—C1	1.246 (3)	C13—H13	0.9300
N2—C17	1.346 (3)	C8—C9	1.377 (4)
N2—C16	1.383 (4)	C8—C10	1.378 (5)
N2—C18	1.474 (3)	C10—C9^iii^	1.378 (4)
C16—C15	1.395 (4)	C10—H10	0.9300
C16—C11	1.393 (4)	C5—C6	1.387 (4)
C1—C2	1.503 (4)	C12—H12	0.9300
C2—C7	1.381 (4)	C18—H18A	0.9700
C2—C3	1.387 (4)	C18—H18B	0.9700
C3—C4	1.375 (4)	C15—C14	1.378 (5)
C3—H3	0.9300	C15—H15	0.9300
C17—H17	0.9300	C9—C10^iii^	1.378 (4)
O3—C8	1.396 (4)	C9—H9	0.9300
O3—C5	1.397 (3)	C14—H14	0.9300
C11—C12	1.398 (4)	C6—H6	0.9300
C4—C5	1.365 (4)		
			
O2—Co1—O2^i^	153.16 (15)	C3—C4—H4	120.3
O2—Co1—N1	92.67 (9)	C18—C19—C19^ii^	111.6 (3)
O2^i^—Co1—N1	103.95 (9)	C18—C19—H19A	109.3
O2—Co1—N1^i^	103.95 (9)	C19^ii^—C19—H19A	109.3
O2^i^—Co1—N1^i^	92.67 (9)	C18—C19—H19B	109.3
N1—Co1—N1^i^	103.44 (13)	C19^ii^—C19—H19B	109.3
O2—Co1—O1	58.59 (8)	H19A—C19—H19B	108.0
O2^i^—Co1—O1	101.36 (9)	C2—C7—C6	120.4 (3)
N1—Co1—O1	150.83 (8)	C2—C7—H7	119.8
N1^i^—Co1—O1	89.54 (8)	C6—C7—H7	119.8
O2—Co1—O1^i^	101.36 (9)	C12—C13—C14	122.0 (3)
O2^i^—Co1—O1^i^	58.59 (8)	C12—C13—H13	119.0
N1—Co1—O1^i^	89.54 (8)	C14—C13—H13	119.0
N1^i^—Co1—O1^i^	150.83 (8)	C9—C8—C10	120.3 (3)
O1—Co1—O1^i^	91.42 (11)	C9—C8—O3	115.4 (3)
C17—N1—C11	104.9 (2)	C10—C8—O3	124.3 (3)
C17—N1—Co1	126.90 (19)	C9^iii^—C10—C8	120.0 (3)
C11—N1—Co1	124.46 (17)	C9^iii^—C10—H10	120.0
C1—O2—Co1	97.48 (18)	C8—C10—H10	120.0
C1—O1—Co1	82.61 (17)	C4—C5—C6	121.4 (3)
C17—N2—C16	107.1 (2)	C4—C5—O3	119.6 (3)
C17—N2—C18	126.5 (2)	C6—C5—O3	118.9 (3)
C16—N2—C18	126.1 (2)	C13—C12—C11	118.0 (3)
N2—C16—C15	131.9 (3)	C13—C12—H12	121.0
N2—C16—C11	105.5 (2)	C11—C12—H12	121.0
C15—C16—C11	122.6 (3)	N2—C18—C19	114.2 (2)
O1—C1—O2	121.2 (3)	N2—C18—H18A	108.7
O1—C1—C2	120.7 (2)	C19—C18—H18A	108.7
O2—C1—C2	118.1 (2)	N2—C18—H18B	108.7
C7—C2—C3	119.4 (2)	C19—C18—H18B	108.7
C7—C2—C1	121.3 (2)	H18A—C18—H18B	107.6
C3—C2—C1	119.2 (2)	C14—C15—C16	116.4 (3)
C4—C3—C2	120.5 (3)	C14—C15—H15	121.8
C4—C3—H3	119.7	C16—C15—H15	121.8
C2—C3—H3	119.7	C10^iii^—C9—C8	119.7 (3)
N1—C17—N2	113.0 (2)	C10^iii^—C9—H9	120.1
N1—C17—H17	123.5	C8—C9—H9	120.1
N2—C17—H17	123.5	C15—C14—C13	121.4 (3)
C8—O3—C5	116.9 (2)	C15—C14—H14	119.3
N1—C11—C16	109.5 (2)	C13—C14—H14	119.3
N1—C11—C12	131.0 (3)	C7—C6—C5	118.8 (3)
C16—C11—C12	119.5 (3)	C7—C6—H6	120.6
C5—C4—C3	119.4 (3)	C5—C6—H6	120.6
C5—C4—H4	120.3		
			
O2—Co1—N1—C17	114.1 (2)	C16—N2—C17—N1	1.6 (3)
O2^i^—Co1—N1—C17	−44.6 (3)	C18—N2—C17—N1	175.4 (3)
N1^i^—Co1—N1—C17	−140.8 (3)	C17—N1—C11—C16	−0.2 (3)
O1—Co1—N1—C17	104.9 (3)	Co1—N1—C11—C16	159.27 (18)
O1^i^—Co1—N1—C17	12.8 (2)	C17—N1—C11—C12	−179.7 (3)
O2—Co1—N1—C11	−40.8 (2)	Co1—N1—C11—C12	−20.2 (4)
O2^i^—Co1—N1—C11	160.4 (2)	N2—C16—C11—N1	1.1 (3)
N1^i^—Co1—N1—C11	64.21 (19)	C15—C16—C11—N1	−178.7 (3)
O1—Co1—N1—C11	−50.1 (3)	N2—C16—C11—C12	−179.3 (2)
O1^i^—Co1—N1—C11	−142.2 (2)	C15—C16—C11—C12	0.9 (4)
O2^i^—Co1—O2—C1	−48.23 (18)	C2—C3—C4—C5	0.0 (4)
N1—Co1—O2—C1	−177.02 (19)	C3—C2—C7—C6	1.0 (4)
N1^i^—Co1—O2—C1	78.4 (2)	C1—C2—C7—C6	177.9 (3)
O1—Co1—O2—C1	−2.29 (17)	C5—O3—C8—C9	156.8 (3)
O1^i^—Co1—O2—C1	−86.95 (19)	C5—O3—C8—C10	−26.4 (5)
O2—Co1—O1—C1	2.31 (17)	C9—C8—C10—C9^iii^	0.6 (6)
O2^i^—Co1—O1—C1	162.98 (16)	O3—C8—C10—C9^iii^	−176.1 (3)
N1—Co1—O1—C1	13.2 (3)	C3—C4—C5—C6	−1.0 (4)
N1^i^—Co1—O1—C1	−104.39 (17)	C3—C4—C5—O3	−178.4 (2)
O1^i^—Co1—O1—C1	104.77 (18)	C8—O3—C5—C4	−86.4 (4)
C17—N2—C16—C15	178.2 (3)	C8—O3—C5—C6	96.1 (4)
C18—N2—C16—C15	4.4 (5)	C14—C13—C12—C11	0.6 (5)
C17—N2—C16—C11	−1.6 (3)	N1—C11—C12—C13	178.3 (3)
C18—N2—C16—C11	−175.4 (3)	C16—C11—C12—C13	−1.1 (4)
Co1—O1—C1—O2	−3.8 (3)	C17—N2—C18—C19	40.2 (4)
Co1—O1—C1—C2	175.3 (2)	C16—N2—C18—C19	−147.2 (3)
Co1—O2—C1—O1	4.4 (3)	C19^ii^—C19—C18—N2	179.41 (19)
Co1—O2—C1—C2	−174.77 (19)	N2—C16—C15—C14	−179.9 (3)
O1—C1—C2—C7	152.1 (3)	C11—C16—C15—C14	−0.1 (4)
O2—C1—C2—C7	−28.8 (4)	C10—C8—C9—C10^iii^	−0.6 (6)
O1—C1—C2—C3	−31.1 (4)	O3—C8—C9—C10^iii^	176.4 (3)
O2—C1—C2—C3	148.1 (3)	C16—C15—C14—C13	−0.4 (5)
C7—C2—C3—C4	0.0 (4)	C12—C13—C14—C15	0.2 (5)
C1—C2—C3—C4	−176.9 (2)	C2—C7—C6—C5	−2.0 (5)
C11—N1—C17—N2	−0.8 (3)	C4—C5—C6—C7	2.0 (5)
Co1—N1—C17—N2	−159.67 (18)	O3—C5—C6—C7	179.4 (3)

Symmetry codes: (i) −*x*+1, *y*, −*z*+3/2; (ii) −*x*+1, *y*, −*z*+5/2; (iii) −*x*, −*y*, −*z*.
